# Supplementary material for: Ultrafast upconversion superfluorescence with a sub-2.5 ns lifetime at room temperature
Source: Nat Commun. 2024 Nov 14;15:9880. doi: 10.1038/s41467-024-54314-x (PMC11564658; doi:10.1038/s41467-024-54314-x)
Supplement: Supplementary file 1 — Supplementary Information [file 41467_2024_54314_MOESM1_ESM.pdf]

Supplementary Information

Ultrafast upconversion superfluorescence with a sub-2.5 ns lifetime at room temperature

Mengwei Zhou,[1,2,3] Ping Huang,[1,2,3,*] Xiaoying Shang,[1,2,*] Ruihuan Zhang,[1] Wen Zhang,[1,3] Zhiqing Shao,[1,3] Shuo Zhang,[1] Wei Zheng[1,2,3,*], and Xueyuan Chen[1,2,3,*]

[1]*State Key Laboratory of Structural Chemistry, Fujian Key Laboratory of Nanomaterials, and CAS Key Laboratory of Design and Assembly of Functional Nanostructures, Fujian Institute of Research on the Structure of Matter, Chinese Academy of Sciences, Fuzhou 350002, China.*

[2]*Fujian Science & Technology Innovation Laboratory for Optoelectronic Information of China, Fuzhou 350108, China.*

[3]*University of Chinese Academy of Sciences, Beijing 100049, China.*

*To whom correspondence should be addressed, E-mail: huangping09@fjirsm.ac.cn, shangxiaoying@fjirsm.ac.cn, zhengwei@fjirsm.ac.cn or xchen@fjirsm.ac.cn.

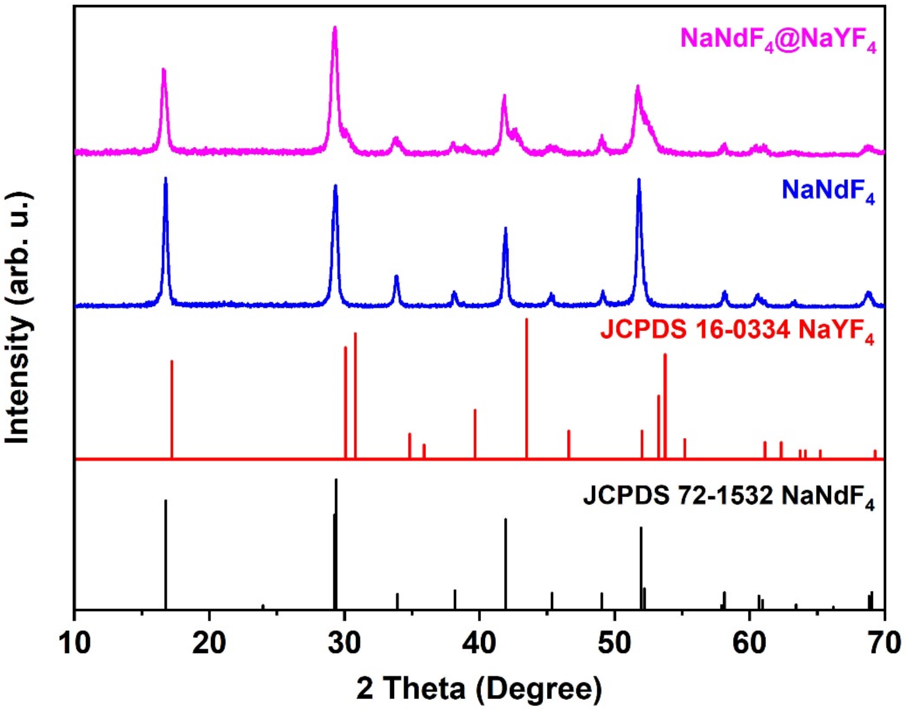

Supplementary Figure 1. Powder X-ray diffraction (XRD) patterns of $NaNdF_4$ core and $NaNdF_4@NaYF_4$ core-shell upconversion nanoparticles (UCNPs), showing pure phase and high crystallinity of the resulting UCNPs.

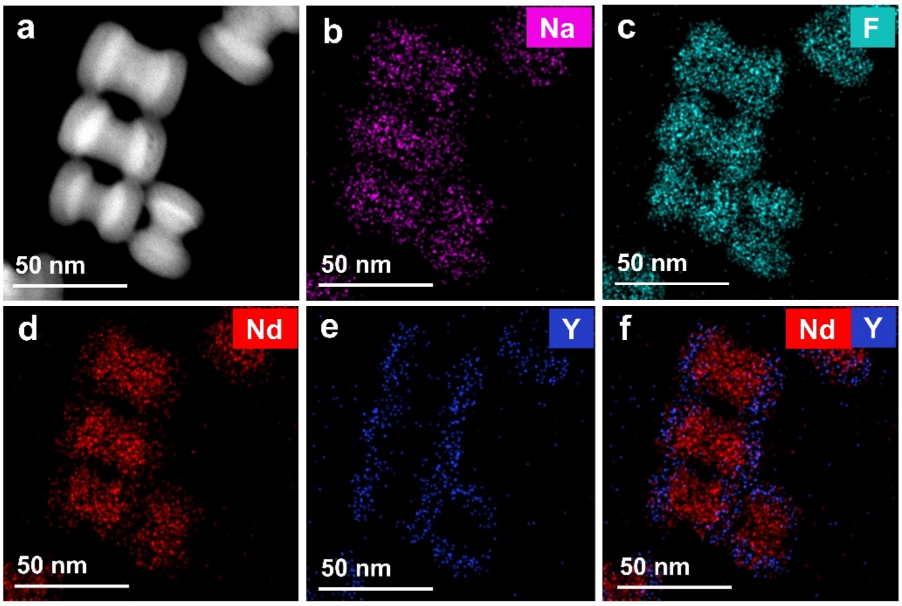

Supplementary Figure 2. (a) High-angle annular dark-field scanning transmission electron microscopy (HAADF-STEM) image and (b-f) energy dispersive X-ray (EDX) elemental mappings of $NaNdF_4$@$NaYF_4$ core-shell UCNPs. The significant difference in the atomic number (Z) contrast and the elemental distributions of Nd and Y demonstrated the core-shell structure of the UCNPs.

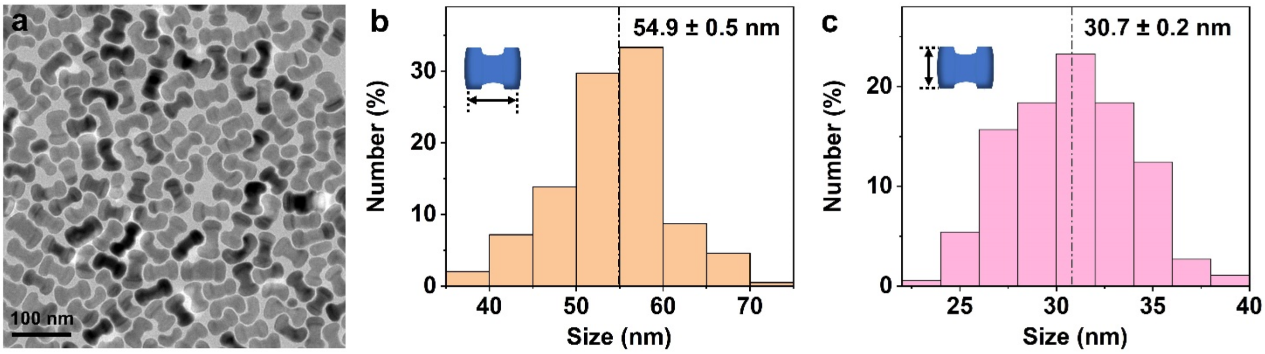

Supplementary Figure 3. (a) TEM image of $NaNdF_4@NaYF_4$ core-shell UCNPs. (b) and (c) show the size distributions of the UCNPs obtained by randomly calculating 100 particles in the TEM image.

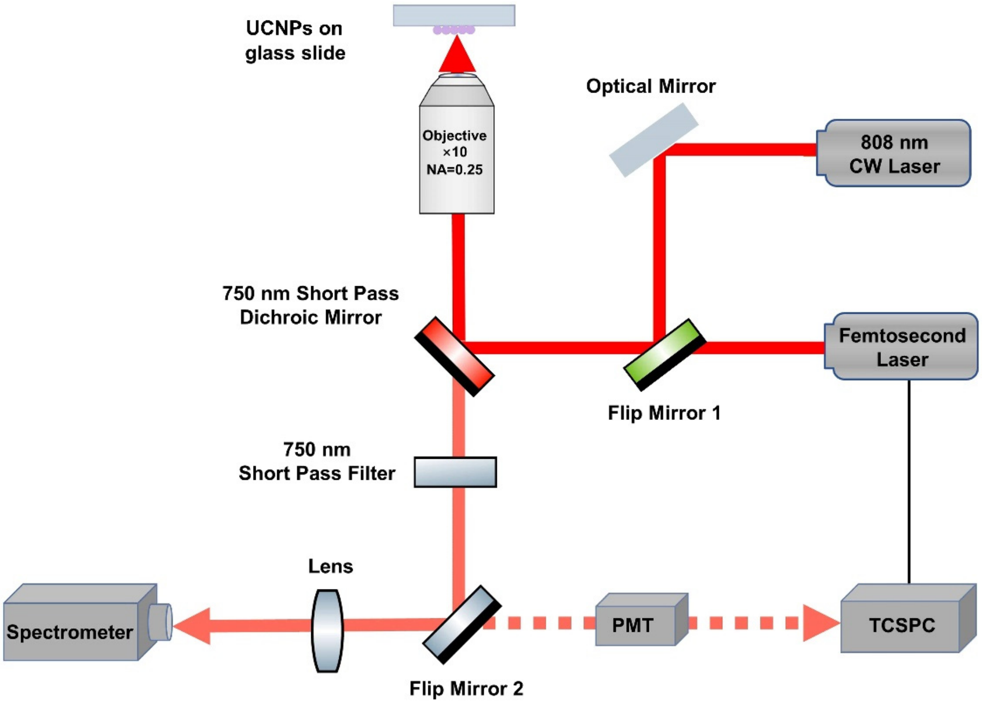

Supplementary Figure 4. Diagram of the customized microscopic spectroscopy system for upconversion superfluorescence (UC-SF) measurements, which was equipped with both continuous-wave (CW) and fs-pulsed laser as two independent excitation sources.

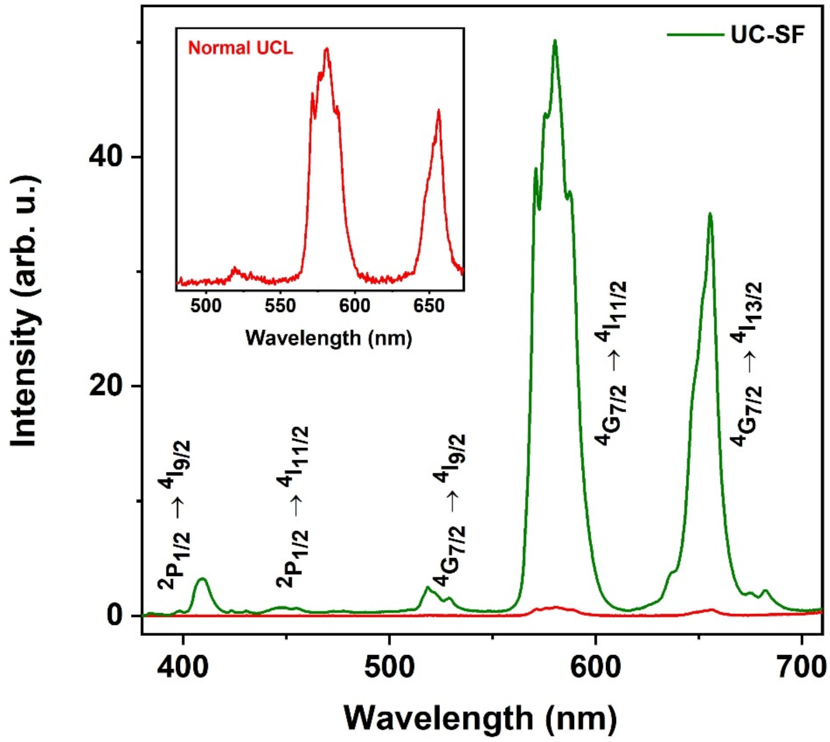

Supplementary Figure 5. Normal upconvension luminescence (UCL) and UC-SF spectra of $NaNdF_4$@$NaYF_4$ UCNPs upon 808-nm CW and 800-nm fs-pulsed laser excitation with an average power density of ~1.10 kW cm^{-2}, respectively. The inset shows the enlarged UCL spectrum.

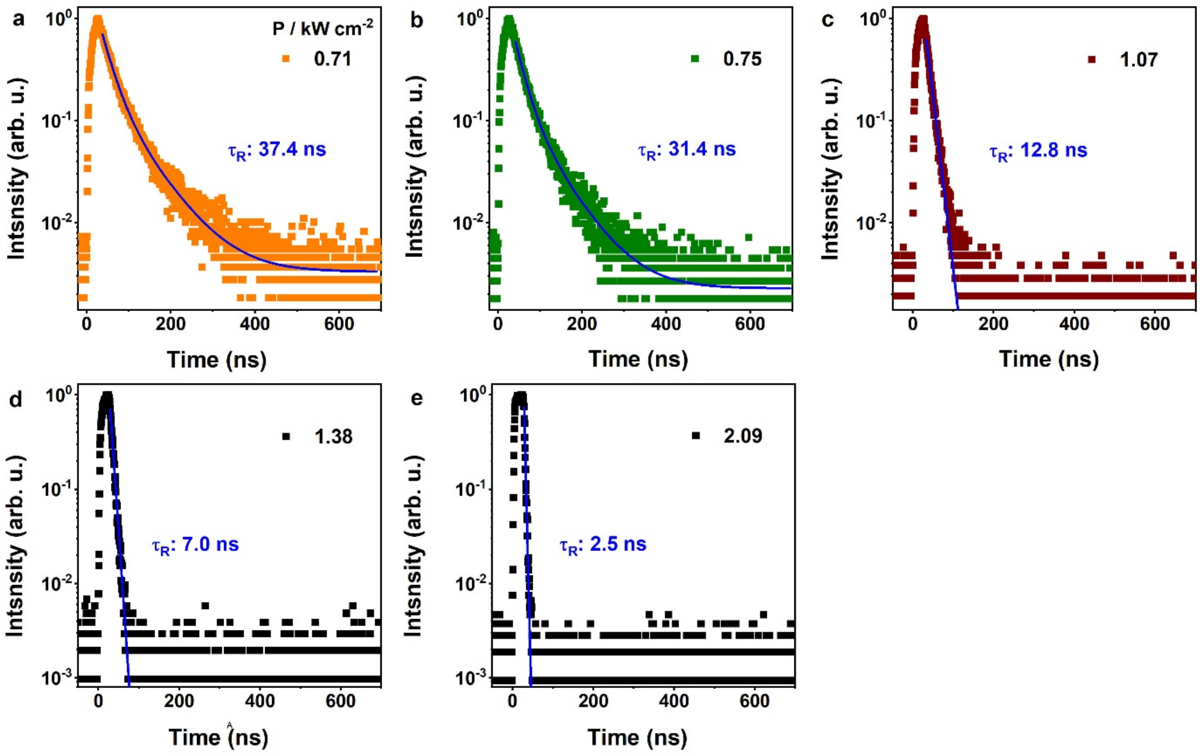

Supplementary Figure 6. Power-dependent UC-SF decay curves (λ_{em} = 588 nm) of $NaNdF_4$@$NaYF_4$ UCNPs upon fs-pulsed laser excitation at 800 nm, showing the decreased radiative decay time (τ_R) with the increasing excitation power density. The blue full lines represent the fitting curves to the decay component. (a,b) At low excitation power densities (0.71−0.75 kW cm^{-2}), the emission of Nd^{3+} deviated from single-exponential decay, due to the mixing of the normal UCL of Nd^{3+}. In this case, the decay curves were fitted with a biexponential function:

$$\mathrm{I}(t) = \mathrm{A}_1\exp\left(-\frac{t}{\tau_1}\right) + \mathrm{A}_2\exp\left(-\frac{t}{\tau_2}\right)$$

where I(t) denotes the luminescence intensity as a function of time; A_1 and A_2 are the weight ratios for the lifetime components of τ_1 and τ_2, respectively. The average decay time of Nd^{3+} was calculated by the following expression:

$$\tau_{ave} = \frac{\mathrm{A}_1 \times \tau_1^2 + \mathrm{A}_2 \times \tau_2^2}{\mathrm{A}_1 \times \tau_1 + \mathrm{A}_2 \times \tau_2}$$

(c-e) At high excitation power densities (1.07−2.09 kW cm^{-2}), the emission of Nd^{3+} turned to pure UC-SF and the decay curves can be fitted with a single-exponential function, whereby the decay time of Nd^{3+} was derived.

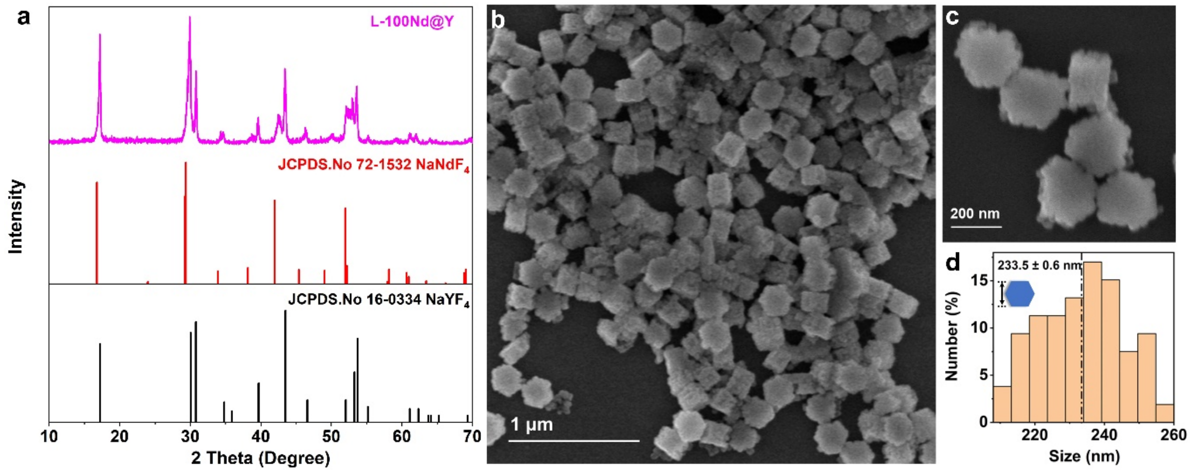

Supplementary Figure 7. (a) XRD pattern of large-size $NaNdF_4$@$NaYF_4$ (L-100Nd@Y) UCNPs. (b) and (c) Scanning electron microscopy (SEM) images of L-100Nd@Y UCNPs. (d) Size distribution of the UCNPs obtained by randomly calculating 100 particles in the SEM image.

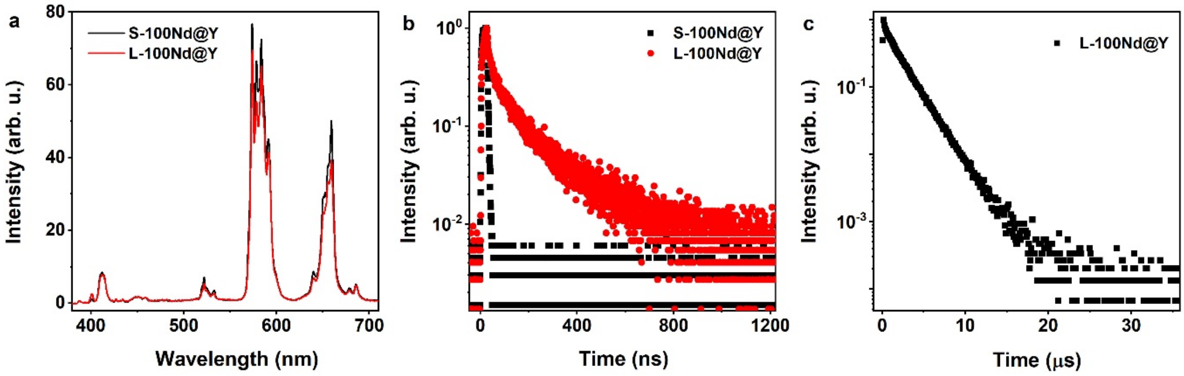

Supplementary Figure 8. (a) UC-SF spectra and (b) decay curves (λ_{em} = 588 nm) of the large-size (~233.5 nm, L-100Nd@Y) and small-size (~54.9 nm, S-100Nd@Y) $NaNdF_4@NaYF_4$ UCNPs, upon fs-pulsed laser excitation at 800 nm with a power density of 2.09 kW cm^{-2}. (c) Normal UCL decay curve of L-100Nd@Y UCNPs by monitoring the $^4G_{7/2} \rightarrow {}^4I_{11/2}$ emission of Nd^{3+} at 588 nm upon excitation with a ns-pulsed laser at 808 nm (10 Hz, pulse width ≤5 ns). The UC-SF decay time (τ_{SF}) of L-100Nd@Y was determined to be 92.8 ns, which is much longer than that (2.5 ns) of S-100Nd@Y, indicating smaller number of coherently coupled dipoles (N) for the $^4G_{7/2} \rightarrow {}^4I_{11/2}$ transition of Nd^{3+} in L-100Nd@Y than in S-100Nd@Y. By single-exponential fitting to the decay curve in (c), the spontaneous decay time (τ_{SE}) from $^4G_{7/2}$ of Nd^{3+} in L-100Nd@Y was derived to be 2.05 μs. According to the equation $\tau_{SF} \propto \tau_{SE}/N$, the number of coherently coupled dipoles (N) for the $^4G_{7/2} \rightarrow {}^4I_{11/2}$ transition of Nd^{3+} in L-100Nd@Y was estimated to be ~23, which is much lower than that (~912) in S-100Nd@Y. Nonetheless, it is significantly improved in comparison with that (N = 11) reported by Lim and Han *et al.* under excitation with a high-repetition (1k Hz) ns-pulsed laser. These observations demonstrate that the improved UC-SF properties achieved in our 100Nd@Y UCNPs lie in the use of small-sample SF system along with fs-pulsed laser excitation, which resulted in significantly enhanced radiation field and consequently increased number of coherently coupled dipoles as compared to those in larger samples with ns-pulsed laser excitation.

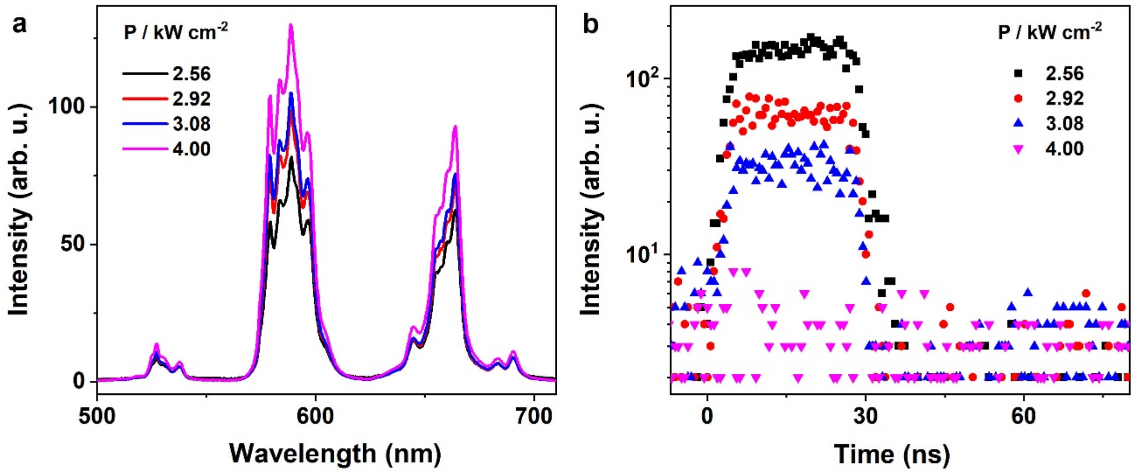

Supplementary Figure 9. Power-dependent (a) UC-SF spectra and (b) decay curves (λ_{em} = 588 nm) of $NaNdF_4$@$NaYF_4$ UCNPs upon 800-nm fs-pulsed laser excitation. The UC-SF intensity continued to increase with increasing the excitation power density beyond 2.56 kW cm^{-2}, because of the increased number of coupled dipoles in the coherent state under high-power excitation. However, the transient UC-SF intensity in the time window of 0−30 ns decreased upon increasing the excitation power density, indicating the shortened decay time of the UCNPs below 2.5 ns that is beyond the detection limit of our instrument.

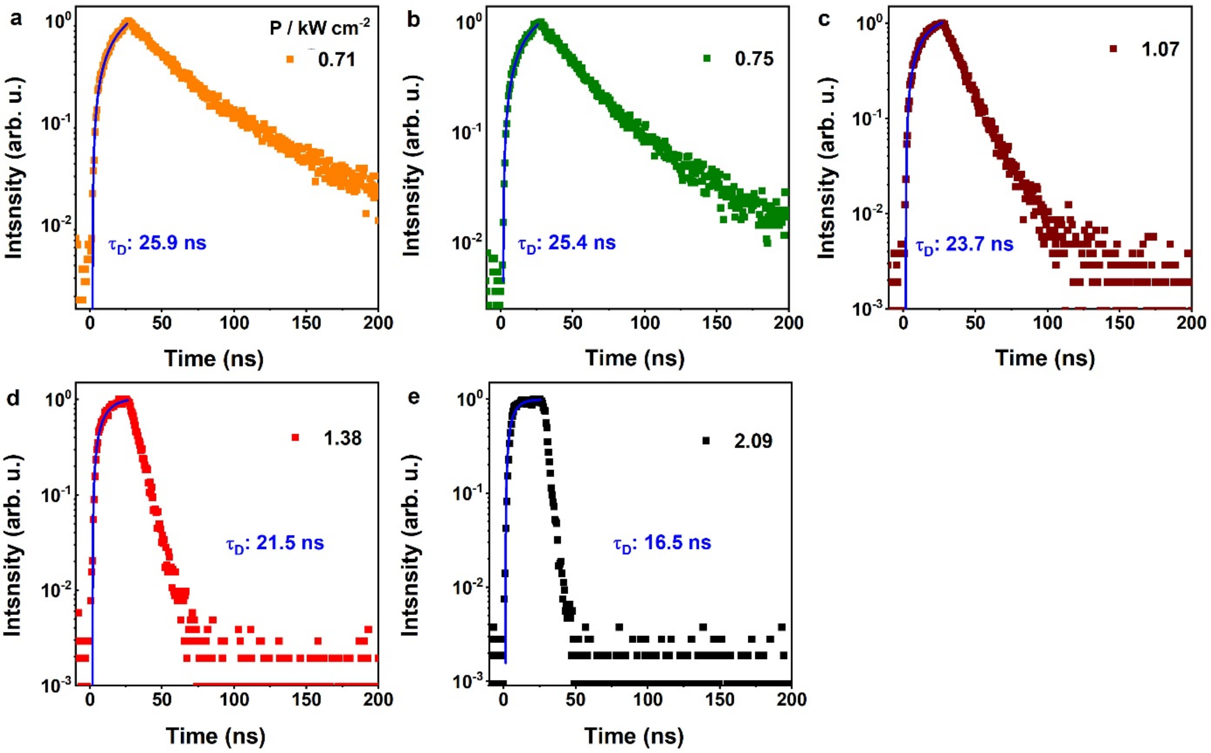

Supplementary Figure 10. (a-e) Power-dependent UC-SF decay curves (λ_{em} = 588 nm) of $NaNdF_4@NaYF_4$ UCNPs at the initial stage upon fs-pulsed laser excitation at 800 nm, showing the decreased delay time (τ_D) with the increasing excitation power density. The blue full lines represent the single-exponential fitting to the rise component of the decay curves and the delay time was determined as 95% of the asymptotic value.

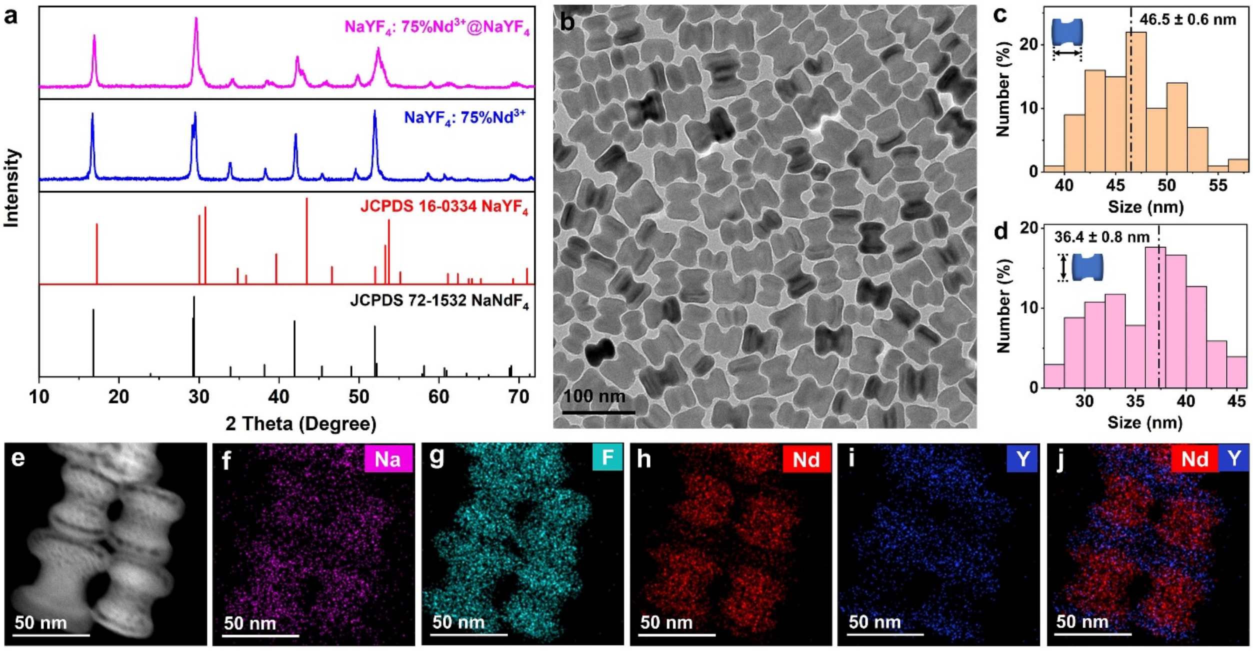

Supplementary Figure 11. XRD patterns of $NaYF_4$: 75%Nd^{3+} core and $NaYF_4$: 75%Nd^{3+}@$NaYF_4$ core-shell UCNPs. (b) TEM image of $NaYF_4$: 75%Nd^{3+}@$NaYF_4$ core-shell UCNPs. (c) Length and (d) diameter distributions of the UCNPs obtained by randomly calculating 100 particles in the TEM image. (e) HAADF-STEM image and (f-j) EDX elemental mappings of $NaYF_4$: 75%Nd^{3+}@$NaYF_4$ core-shell UCNPs.

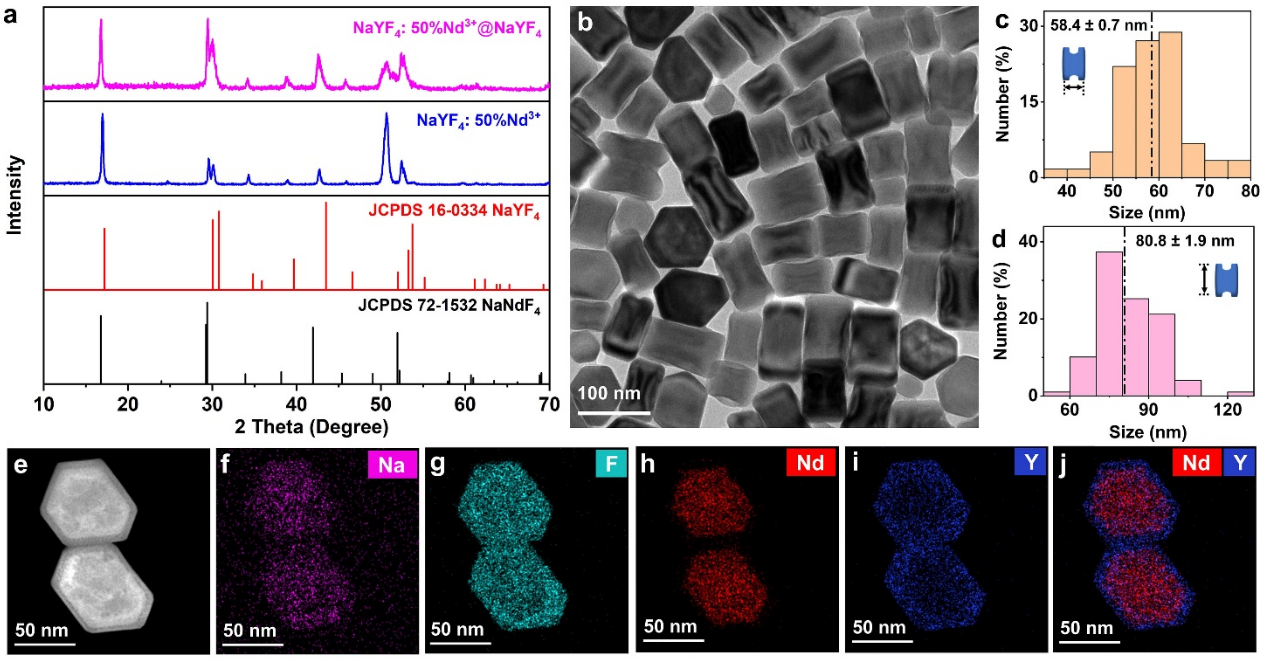

Supplementary Figure 12. (a) XRD patterns of $NaYF_4$: 50%Nd^{3+} core and $NaYF_4$: 50%Nd^{3+}@$NaYF_4$ core-shell UCNPs. (b) TEM image of $NaYF_4$: 50%Nd^{3+}@$NaYF_4$ core-shell UCNPs. (c) Length and (d) diameter distributions of the UCNPs obtained by randomly calculating 100 particles in the TEM image. (e) HAADF-STEM image and (f-j) EDX elemental mappings of $NaYF_4$: 50%Nd^{3+}@$NaYF_4$ core-shell UCNPs.

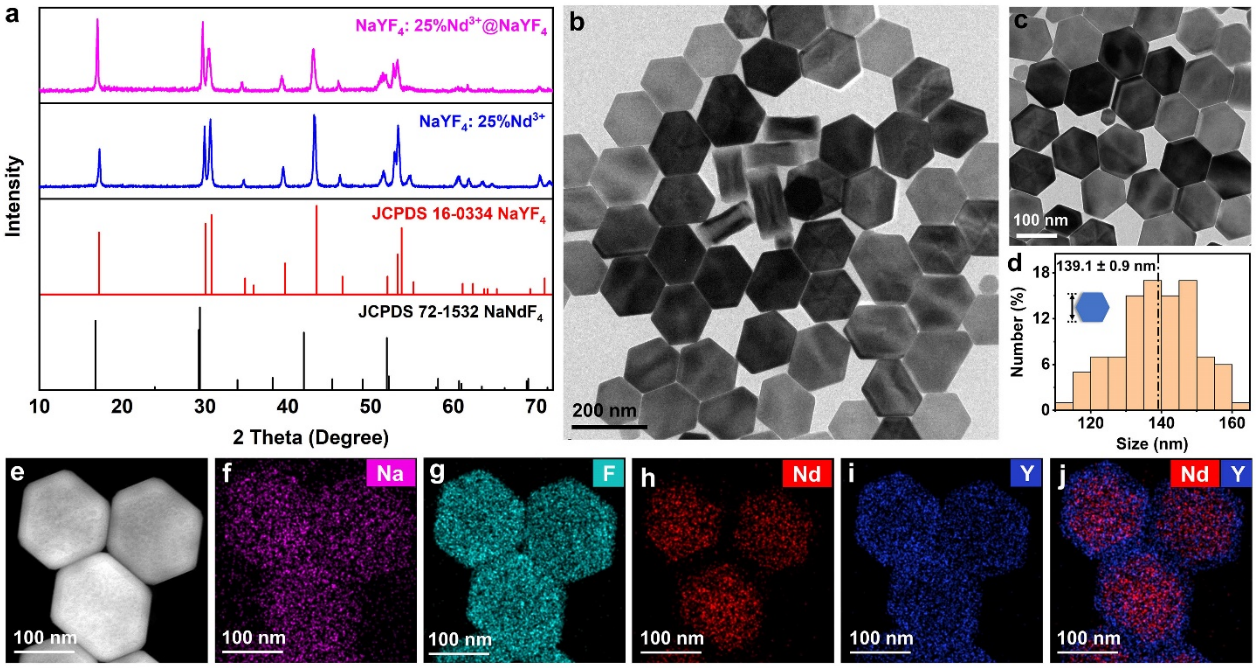

Supplementary Figure 13. (a) XRD patterns of $NaYF_4$: 25%Nd^{3+} core and $NaYF_4$: 25%Nd^{3+}@$NaYF_4$ core-shell UCNPs. (b) and (c) TEM images of $NaYF_4$: 25%Nd^{3+}@$NaYF_4$ core-shell UCNPs. (d) Size distribution of the UCNPs obtained by randomly calculating 100 particles in the TEM image. (e) HAADF-STEM image and (f-j) EDX elemental mappings of $NaYF_4$: 25%Nd^{3+}@$NaYF_4$ core-shell UCNPs.

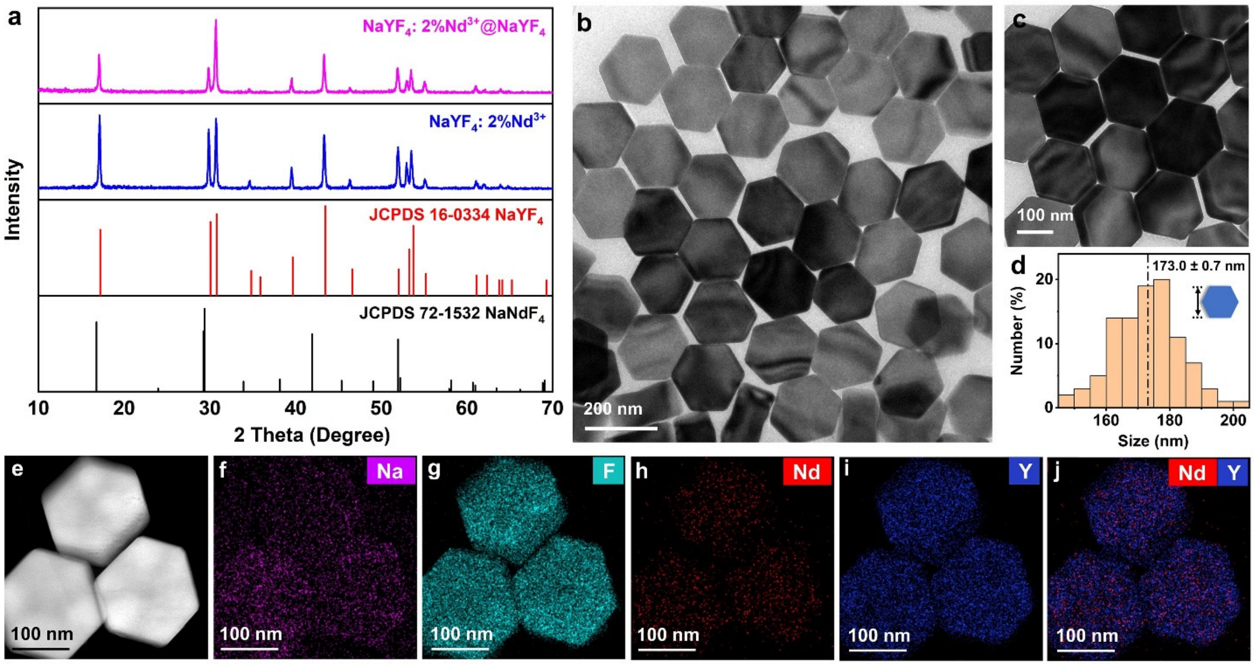

Supplementary Figure 14. (a) XRD patterns of $NaYF_4$: 2%Nd^{3+} core and $NaYF_4$: 2%Nd^{3+}@$NaYF_4$ core-shell UCNPs. (b) and (c) TEM images of $NaYF_4$: 2%Nd^{3+}@$NaYF_4$ core-shell UCNPs. (d) Size distribution of the UCNPs obtained by randomly calculating 100 particles in the TEM image. (e) HAADF-STEM image and (f-j) EDX elemental mappings of $NaYF_4$: 2%Nd^{3+}@$NaYF_4$ core-shell UCNPs.

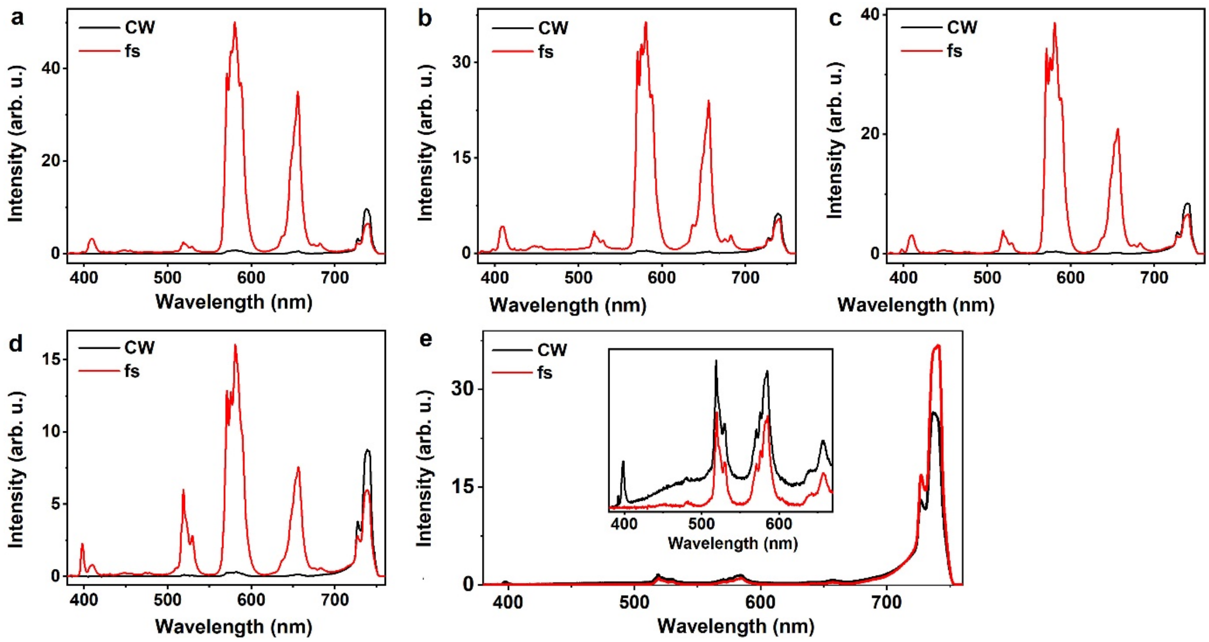

Supplementary Figure 15. Comparation of normal UCL and UC-SF of (a) $NaNdF_4$@$NaYF_4$, (b) $NaYF_4$: 75%Nd^{3+}@$NaYF_4$, (c) $NaYF_4$: 50%Nd^{3+}@$NaYF_4$, (d) $NaYF_4$: 25%Nd^{3+}@$NaYF_4$, and (e) $NaYF_4$: 2%Nd^{3+}@$NaYF_4$ core-shell UCNPs upon 808-nm CW laser and 800-nm fs-pulsed laser excitation, respectively, with an equivalent power density at average (~1.10 kW cm^{-2}).

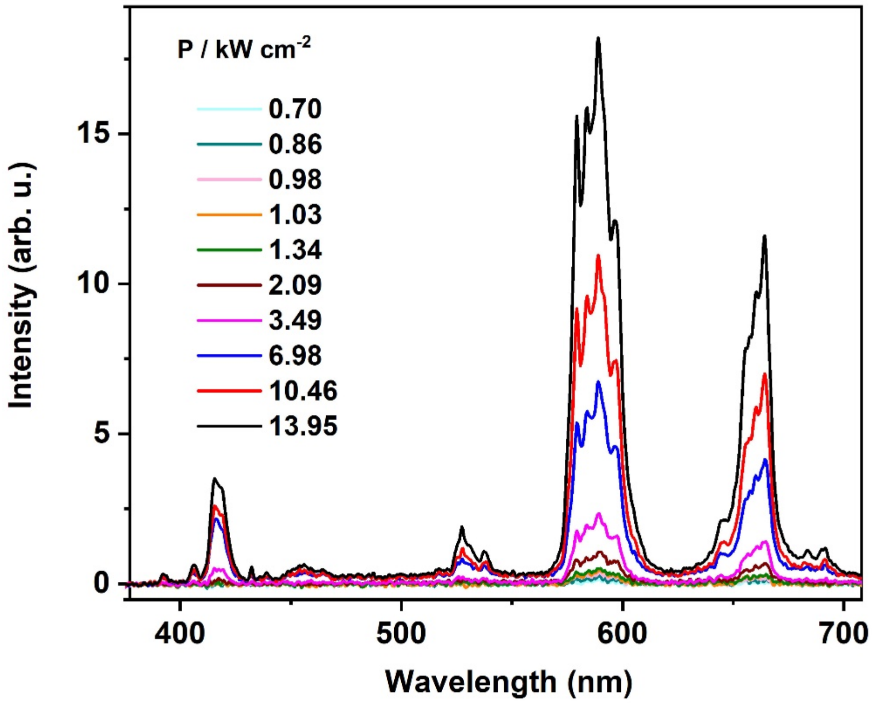

Supplementary Figure 16. Power-dependent UC-SF spectra of $NaYF_4$: 50%Nd^{3+}@$NaYF_4$ core-shell UCNPs upon fs-pulsed laser excitation at 800 nm.

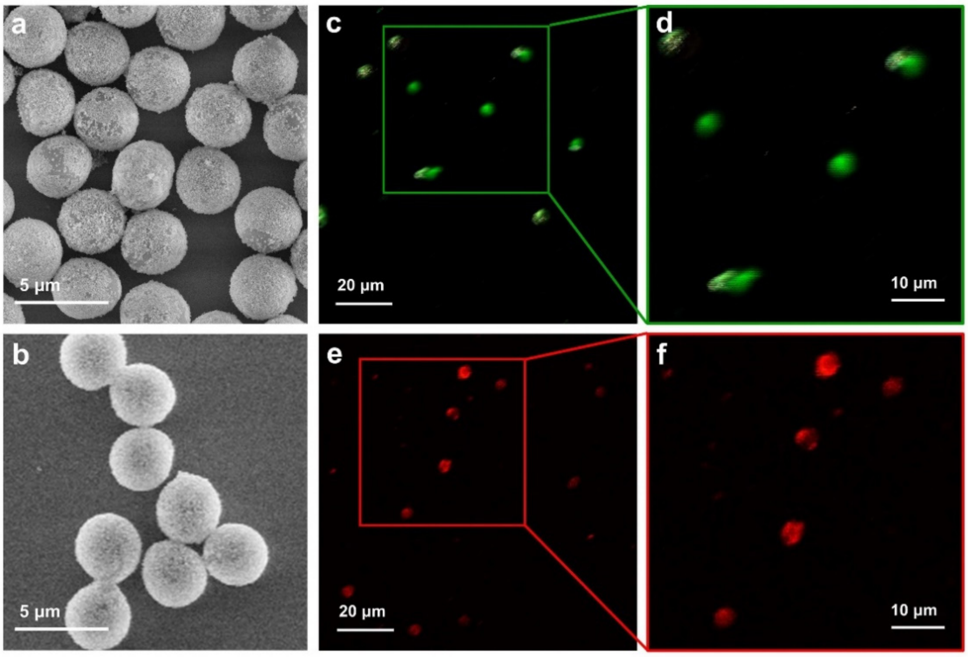

Supplementary Figure 17. SEM images of (a) 2Er@Y and (b) 100Nd@Y UCMSs. Laser scanning microscopy images of (c,d) 2Er@Y and (e,f) 100Nd@Y UCMSs, collected in the green (500-550 nm) and red channel (570-620 nm), respectively. Image dimensions: 1024 × 1024 pixels; pixel size: 0.11 μm; pixel dwell time: 1.1 μs; acquisition time: 16.6 s. When the pixel dwell time was set as 1.1 μs, the images of 2Er@Y UCMSs suffered from an obvious tailing effect due to the μs−ms long radiative lifetime of normal UCL, which caused the image distortion and deformation. For comparison, the images of 100Nd@Y UCMSs were clear and free from tailing effect because of the ultra-short decay time (on the ns scale) of UC-SF.
